# A System Biology-Based Approach for Designing Combination Therapy in Cancer Precision Medicine

**DOI:** 10.1155/2020/5072697

**Published:** 2020-08-26

**Authors:** S. H. Sabzpoushan

**Affiliations:** Department of Biomedical Engineering, Iran University of Science and Technology (IUST), Tehran 16846-13114, Iran

## Abstract

In this paper, we have used an agent-based stochastic tumor growth model and presented a mathematical and theoretical perspective to cancer therapy. This perspective can be used to theoretical study of precision medicine and combination therapy in individuals. We have conducted a series of *in silico* combination therapy experiments. Based on cancer drugs and new findings of cancer biology, we hypothesize relationships between model parameters which in some cases represent individual genome characteristics and cancer drugs, i.e., in our approach, therapy players are delegated by biologically reasonable parameters. *In silico* experiments showed that combined therapies are more effective when players affect tumor via different mechanisms and have different physical dimensions. This research presents for the first time an algorithm as a theoretical viewpoint for the prediction of effectiveness and classification of therapy sets.

## 1. Introduction

Study of cancer as the second leading cause of human mortality is essential. Early diagnosis and appropriate therapies can be a significant help to the improvement of cancer survivals. Although surgery in the case of solid tumors, antitumor drugs, radiation, and immunotherapy have been the treatment of choice in some instances, but ineffectiveness of treatments, drug resistance, side effects of therapies, and tailoring treatment to the individual characteristics of each patient are still major clinical problems. Where precision medicine will allow researchers to predict more accurately which therapies will work better in which groups of people, combination therapy is a keystone of cancer therapy and potentially reduces drug resistance, while simultaneously providing therapeutic anticancer benefits, such as reducing tumor growth and metastatic potential, arresting mitotically active cells, reducing cancer stem cell populations, and inducing apoptosis.

A high percent of oncology drugs and therapies fails in clinical trials [1]. This imposes extra expenses to patients and causes the loss of time in cancer therapies. Mathematical models, *in silico* experiments, and simulations can be a great help for evaluation of different therapies and examining diverse strategies of drug therapies.

Ten major characteristics of cancer, known as cancer hallmarks, have been universally recognized as (1) unlimited multiplication, (2) evasion from growth suppressors, (3) promoting invasion and metastasis, (4) resisting apoptosis, (5) stimulating angiogenesis, (6) maintaining proliferative signaling, (7) elimination of cell energy limitation, (8) evading immune destruction, (9) genome instability and mutation, and (10) tumor-enhanced inflammation [2]. Regarding above hallmarks, it is plausible that we attribute drug therapy efficiencies to individuals' genome, i.e., individual's heterogeneity should be taken into account in cancer therapies by some means [3].

Efforts have been devoted to determine how cellular and noncellular components of the tumor's surrounding environment may help it to acquire these characters. This environment and its cellular and noncellular components are called tumor microenvironment (TME) [4–7]. The recognition that cancer cells need their microenvironment to efficiently display their phenotype has opened the door to hypothesize and implement new therapeutic strategies.

Today, the main tumor therapy strategies consist of surgery, radiological intervention, chemotherapy, and somatostatin analogs to control symptoms. However, it seems that tumor cells are particularly clever and elastic, and may adapt to treatments and environmental modifications quickly, i.e., once one component has been blocked, other mechanisms will rapidly follow. This may be one of the main factors that lead to poor cancer therapies [8]. It is why different obstructing mechanisms at the same time might lead to the best results of tumor development prevention [9]. The above facts illuminate the motivation for researches in the field of combination therapies.

Precision medicine refers to the tailoring of medical treatment to the individual characteristics of each patient. It often involves the application of system biology to analyze the cause of an individual patient's disease at the molecular level and then to utilize targeted treatments (often combinatorial) to address that individual patient's disease process. The branch of precision medicine that addresses cancer is referred to as “precision oncology” [3, 10–12].

Tumors are encircled by extracellular matrix (ECM) and stromal cells, and the physiological state of the TME is closely connected to every step of tumorigenesis. Evidence suggests that the vital components of the TME are (1) fibroblasts and myofibroblasts, (2) neuroendocrine cells, (3) adipose cells, (4) immune and inflammatory cells, (5) the blood and lymphatic vascular networks, and (6) extracellular matrix (ECM) [13].

The combinatorial complexity of possible combination therapies [14] and the expense and risks of trial and error experiments, as well as the lack of time for cancer patients, are the main reasons for combination therapies fail in clinical trials. In this circumstance, appropriate biologically realizable models and bioinformatics can be a solution. Mathematical models and computer simulations can be good alternatives for preestimations and evaluations of effectiveness of drug therapies strategies. Computational oncology [15] and *in silico* trials are good preclinical alternatives to predict the progress of the disease in individuals and suggest new diagnostic and therapeutic methods.

Patients with cancer are known to be at an increased risk for community-acquired respiratory viruses, such as SARS CoV-2. There is high proportion of patients who acquired the infection while already in the hospital for cancer treatment affairs. Using bioinformatics, mathematical and computational models and *in silico* analysis are very safe and cost-effective tools for design and analyzing therapy strategies. *In silico* trials as precision medicine simulators can reduce patient commuters to hospitals and high-risk health centers [16].

Cancerous system models can be categorized into three general groups: continuous, discrete, and hybrid, where each one may have deterministic or stochastic formalisms. Continuous models describe the system by using ordinary differential equations (ODEs) or partial differential equations (PDEs). Several researchers have used ODEs to study the growth of tumors. To capture spatial structures of tumors, one should use PDEs. PDEs can better express the temporal and spatial properties of tumor growth at the same time [17–19].

In the family of agent-based models (ABMs), cells are considered as discrete elements, and the interaction between them is defined by biological-based rules [20–26]. ABMs can simulate emergent structures, i.e., structures that a number of not too complex components work together and form more complex behaviors as a group. It is noteworthy that in differential equation- (DE-) based models, rules are applied to the whole system where in ABMs, the functional rules of each single agent can be specific and special to that agent. These make ABMs more appropriate for emergent behaviors like tumor growth and TME dynamics modeling. ABM is also one of the most frequently used methods for modeling multiscale systems like cancers [27].

Today, cancer therapy has dramatically changed, i.e., surgery and radiotherapy are not the only effective ways to fight tumor. Novel methods and approaches are emerging, where the molecular and agent features of tumors seem to be the keystone of any therapy. New antibodies, small molecules, antiangiogenics, viral therapy, and precision medicine methods are typical examples. Because of the abovementioned new therapies use microscopic or molecular level agents explicitly, so system biology-based cancer models can be the most satisfactory candidates for *in silico* experiments and studies.

It is notable to remember that system biology is a term to describe the study of the interactions between the components of the biological systems and how these interactions give rise to the function and behavior of that system. However, although system biology-based mathematical models of cancer are very useful, but they have also limitations, because the recognition of the mechanisms governing cancerous systems has practical limitations as well [17, 18].

In this paper, we use our recently published agent-based stochastic tumor growth model (ABSM) as a cancer system. We design several combination therapies which can be hypothesized and regulated as precision medicine. We postulate five novel quantitative merits for comparing possible effectiveness of different combination therapies. We show how *in silico* experiments can help oncologists to conduct and design combination therapies, and test their ideas, considering some parameters as individual-dependent and tailor more effective medical treatments.

## 2. Materials and Method

In this research, we have used our previously proposed ABSM model [28] as a cancer system, i.e., we may fit it to a given patient and use it for demonstrating our system biology-based approach for designing combination therapy in cancer precision medicine.

**Figure 1:**
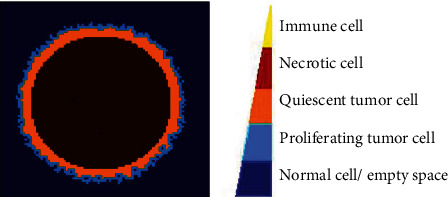
In ABSM, a multicellular tumor consists of an outer shell of proliferating tumor cells (light blue), an inner layer of quiescent tumor cells (orange) which are dormant but viable, and a central core of necrotic entities (dark red). Normal cells are shown in dark blue.

Agent-based modeling is a stochastic approach used to describe a population of interacting agents, where agents behave according to a set of rules that represent the dynamic features of system. In this way, our ABSM has been established on four bases: (1) biological assumptions, (2) physical structure, (3) agents and their states, and (4) states transition rules. The multilayer structure of the ABSM is shown in Figure 1.

In ABSM, host tissue is assumed as a two-dimensional lattice composed of *n*cell × *n*cell squares as illustrated in Figure 10. Here, each square of the lattice is called a cell.

In the ABSM, two types of agents (immune (IA) and nonimmune (NA) cells) are presumed, where three types of NAs (normal cell or empty space (NA_0), proliferating tumor cell (NA_1), nonproliferating or quiescent cell (NA_2), and necrotic cell (NA_3)) are considered.

In the ABSM, we assume two types of NA_1 cells with different division probabilities: in the first type denoted by NA_1_1, the cell division is not influenced by its neighbors, while in the second symbolized by NA_1_2, the cell division probability (*ρ*_PT_) is affected by its healthy neighbors (NA_0s).

In the ABSM, it is assumed that each NA_1_1 may be divided into one NA_1_1 and one NA_1_2 daughter cells with the probability *Nmm* or into two NA_1_1 daughter cells with the probability (1 − *Nmm*). It means the higher the value of *Nmm*, the more susceptibility of cancerous cell division to its microenvironment.

In Table 1, the basic elements of ABSM and their brief descriptions are summarized. As it was stated beforehand, the ABSM is a system biology-oriented model in the sense that it is constructed from several agents and interaction rules between them, and as is shown in [28], these elements and interactions can give rise to the function and behavior of cancer growth system.

With a conceptual and intuitive look at the elements of Table 1, some of them can be assumed individual-dependent, those are indicated by a star mark. You see all but one of them has probability dimensions. We will discuss more this matter in following sections.

More details and descriptions about the ABSM can be found in Appendix A and [28] as well.

### 2.1. Combination Therapy and Precision Medicine

Combination therapy, i.e., a treatment approach that combines two or more therapeutic agents, is a keystone of cancer therapy. The consolidation of anticancer drugs and therapies enhances efficiency compared to the monotherapy approach, because it targets key pathways in a characteristically synergistic or an additive manner. This approach potentially reduces drug resistance, while simultaneously providing therapeutic anticancer benefits, such as reducing tumor growth and metastatic potential, arresting mitotically active cells, reducing cancer stem cell populations, and inducing apoptosis [15, 29].

In this section, we perform *in silico* experiments as preclinical tests to design and suggest combination therapies with the use of ABSM.

Based on cancer drugs and new findings of cancer biology, we hypothesize relations between model parameters and cancer drugs, and therapies, i.e., we assume each drug in a target group can impact related parameter(s) and show by controlling combination of drugs (controlling parameters); we may simulate combination therapy strategies and control tumor size.

**Figure 2:**
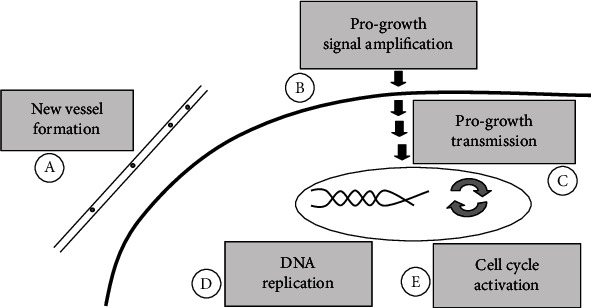
Schematic illustration of five microscopic level targeted therapies.

Having in mind system biology approaches, a survey of cancer therapy literature shows that we may classify five possible groups of target agents in cancer therapies as (A) new vessel formation agents, (B) progrowth signal amplification agents, (C) progrowth signal transmission agents, (D) DNA replication-related agents, and (E) cell cycle activation agents. These groups and their relative levels are schematically illustrated in Figure 2.

Concerning the definitions and roles of the parameters of the ABSM, we have hypothesized the relations of four model parameters, *p*_01_, *p*_02_, age, and *Nmm*, with above groups as is illustrated in Table 2.

In ABSM, the parameters *p*_01_, *p*_02_ are base probabilities of division of NA_1_1 and NA_1_2 cells, respectively, and *Nmm* is the probability of production of a NA_1_2 from the mitosis of a NA_1_1. Age is maximum allowable time duration to stay in NA_1 mode without proliferation. We see *p*_01_, *p*_02_, and *Nmm* are probability numbers; thus, their values can be on [0, 1] interval, where age is time or more precisely number of iterations [28].

Progrowth signal transmission, as is depicted in Figure 2, sends division signals to the cell; after the first molecule in a pathway receives a signal, it activates another molecule; this process is repeated until the last molecule is activated and the cell function is carried out. Therefore, the assumption that age may be considered as a control tool of the target group C is biologically plausible, i.e., “age” can control the delay of progrowth signal.

As a drug in the group B, one may name “trastuzumab”, an antibody drug conjugate (ADC) consisting of the recombinant antiepidermal growth factor receptor 2 (HER2) monoclonal antibody trastuzumab conjugated to the maytansinoid DM1 via a nonreducible thioether linkage (MCC) with potential antineoplastic activity. The trastuzumab moiety of this ADC binds to HER2 on tumor cell surfaces; upon internalization, the DM1 moiety is released and binds to tubulin, thereby disrupting microtubule assembly/disassembly dynamics and inhibiting cell division and the proliferation of cancer cells that overexpress HER2. All of these mean that it is reasonable to relate parameters such as *p*_01_,*p*_02_ that are related to cell division (proliferation) by any means to drugs like *trastuzumab* and treat them as tools for controlling the target groups (B, D, and E).

In ABSM, the parameter *Nmm* is related to proliferative cell type and quality; this is why we have attributed it to the target group D.

On the one hand, all parameters in ABSM have exact mathematical definitions and biological interpretations, and on the other hand, all the abovementioned drugs and many others which are used in cancer therapy [35] are validated clinically [30–34], so we can treat Table 2 as a theoretical deduction which implicitly is supported clinically, i.e., the mapping from treatments to model parameters is implicitly validated.

For simplicity and ease of graphical and qualitative analysis, in this research, we only consider combinations of two target groups for therapies, and because we have no delegated parameter in the group A, so the number of sets of two groups of four groups, B, C, D, and E, will be six as BC, BD, BE, CD, CE, and DE. Reconsidering these six possible combinations and dismissing repeated parameter sets, i.e., combinations that have same players, we consider three distinct combinations of the groups, BC, BD, and CD, with the attributed players as are listed in Table 3.

As we stated earlier, *p*_01_, *p*_02_, and *Nmm* are probability numbers, so they range on the [0, 1] interval, and besides considering their definition in ABSM, they generally represent the probability of (abnormal) proliferation of their respective agents (cells). Therefore, a large value, i.e., near unit, means high probability of cell division where a small value (near zero) means low probability.

Generally speaking, anticancer drugs of the groups B, C, D, and E try to slow cell reproduction via their underlying mechanisms; therefore, it will be plausible if we assume a relation between drug dosage and its effect on cell division probability, i.e., the more the value of drug dosage, the less the value of expected probability value will be. Because maximum allowable dosage of a drug differs from case to case, we consider qualitative measures High (H), Medium (M), and Low (L) and assume that these values act against H, M, and L proliferation (division) probability, respectively. In this situation, we are so lucky because probability number interval is known to be on [0, 1] interval. In Table 4, some typical values are listed, although these values are chosen randomly for simulation investigations, but we may attribute them to individuals' variabilities in genes or biological characteristics and may also attribute them to High, Medium, and Low dosages by some means. This matter will be discussed more in Discussion.

## 3. Results

### 3.1. Group BC Combined Therapies

**Figure 3:**
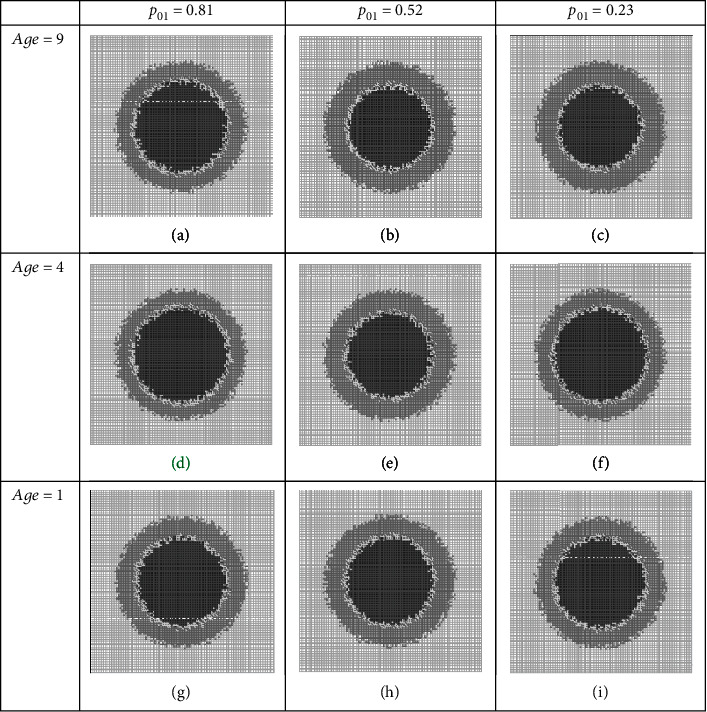
Simulation of the therapy regime which targets progrowth signal amplification and progrowth transmission in a given patient. Snapshots of tumor on day 12 at different dosages (a–i) of a combination therapy regime.

In this strategy of combination therapies, we assume that therapy agents disturb (target) progrowth signal amplification and progrowth transmission at the same time. We assume that the tumor growth system is the same as the one given in Figure 14 (in fact using medical records of a patient, a physician can use ABSM to simulate existing tumor growth in the patient, i.e., run a simulation like Figure 14 and draw a table like Table 15, so she or he can have an estimate of parameters: *p*_01_, *p*_02_, *Nmm*, and age). We see this (assumed) given patient has individual characteristics or personalized genetics as *p*_01_ = 0.7, *p*_02_ = 0.5, *Nmm* = 0.2, and age = 1; we set up *in silico* combination therapy experiments and examine the tumor growth, with controlling the set (*p*_01_, age) via dosages. The results are depicted in Figures 3(a)–3(i). These figures show tumor structural details qualitatively in the day 12th. In all figures, the light grey represents normal tissue cells, the heavy grey represents outer region of tumor that is comprised of proliferating cells, the middle region of white color is nonproliferative (quiescent) cells, and the black region is necrotic cells.  Table 5 compares the tumor structures quantitatively; as you see, we have chosen necrotic fraction (NF), pure tumor growth fraction (PGF), and growth fraction (GF) [28], as quantitative measures to judge the effectiveness of the therapies.

As one sees, the size of tumor in this regime (PGF), on day 12^th^, is a number between 38 and 40 percent of the considered tissue depending on the doses values, where the harmfulness of it (GF) varies between 40 and 55 percent; in this circumstance, the NF measure, that can be assumed as a therapy effectiveness feature after 12 days, is a number between 37 and 52 percent. As we will see later, the best result of therapy is seen in this set; it is highlighted in in Figure 3(d) and Table 5.

**Figure 4:**
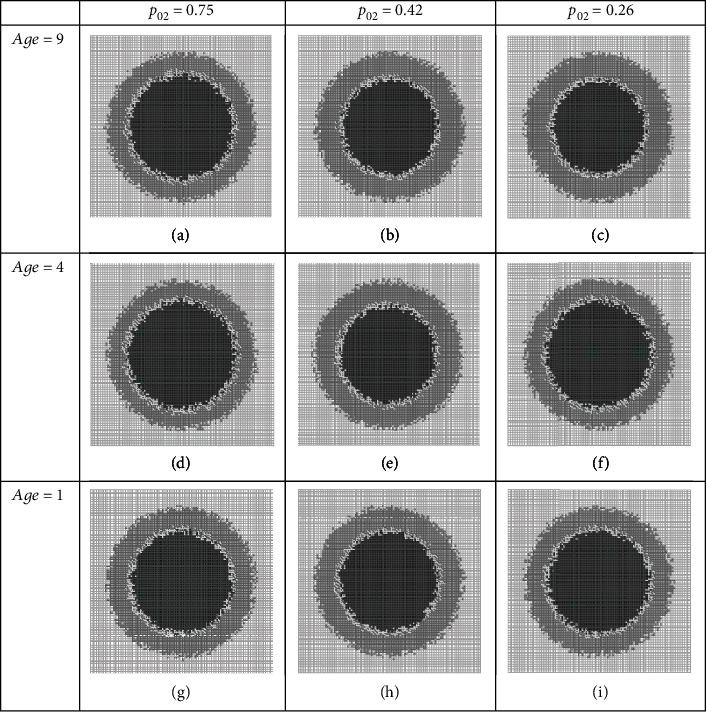
Simulation of a treatment administration which targets progrowth signal amplification and progrowth transmission. Snapshots of cancer system on day 12, at different dosages (a–i) of a combination therapy regime. Quantitative measures are listed in Table 6.

Another possible combination of players in the BC group is (*p*_02_, age). We repeat our *in silico* study as above; the qualitative and quantitative results are depicted in Figures 4(a)–4(i) and Table 6, respectively.

The size of tumor in this regime (PGF), on day 12th, is around 51 percent of the considered tissue, i.e., larger than the previous one. Where the harmfulness of it (GF) varies between 40 and 54 percent, in this condition, NF, that can be assumed as a therapy effectiveness feature after 12 days, is a number between 38 and 51 percent. It seems that this set of players (*p*_02_, age) is weaker than its counterpart (*p*_01_, age) of the BC group in the fight against cancer.

### 3.2. Group BD Combined Therapies

**Figure 5:**
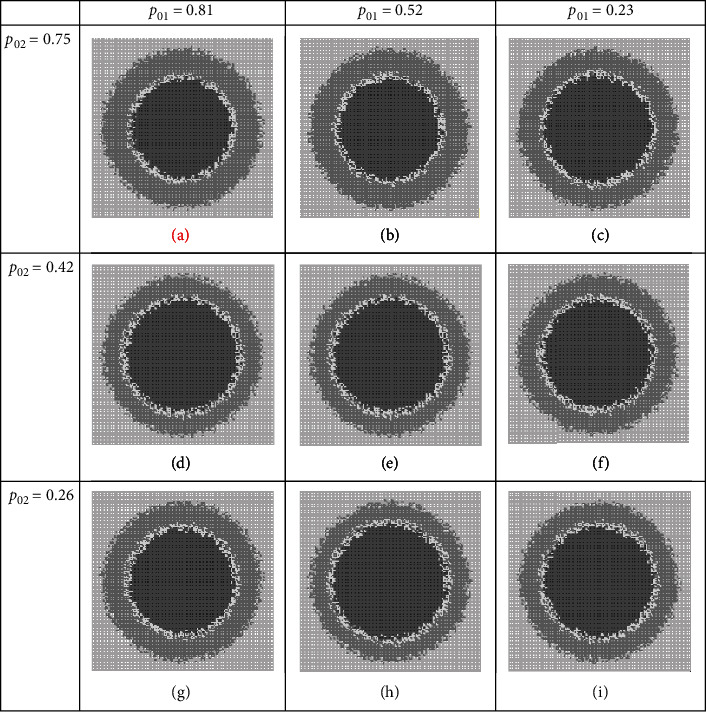
*In silico* experiment results when targeting progrowth signal amplification and DNA replication process. All tumor structures on day 12, but at different dosages (a–i) of a combination therapy regime. Quantitative measures are listed in Table 7.

**Figure 6:**
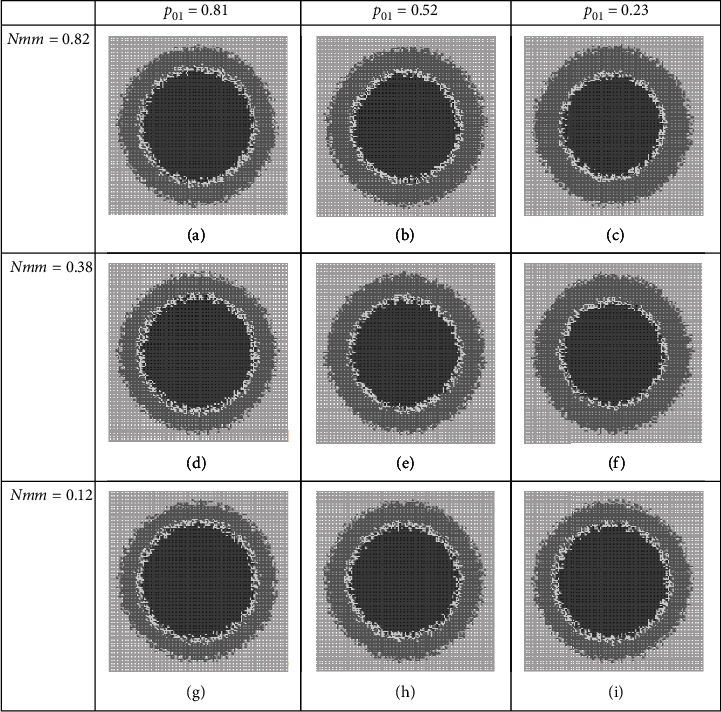
Snapshots of tumor structure where therapy regime hits progrowth signal amplification and DNA replication process. All tumor structures of cancer system on day 12, but at different dosages (a–i) of a combination therapy.

**Figure 7:**
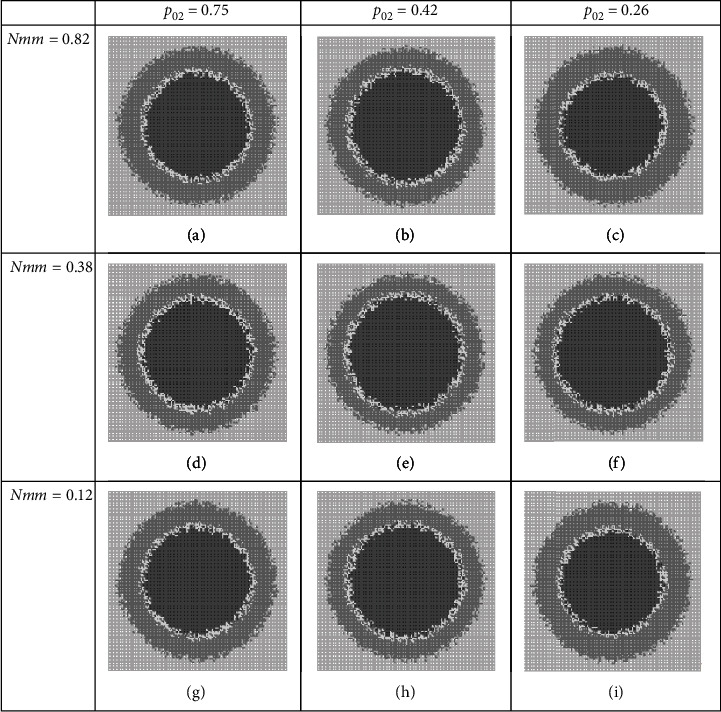
Tumor structure of hypothesized cancer system on day 12, at different dosages (a–i) of a combination therapy regime. This regime targets progrowth signal amplification and DNA replication process. Quantitative measures are listed in Table 9.

In this tactic of combination therapy, we assume that therapies' players disturb progrowth signal amplification and DNA replication process at the same time. Here again, it is assumed that the tumor growth system is the one that considered in Figure 14. We set up *in silico* combination therapy experiments and examine the tumor growth, where varying the values of dosages (the parameter values in the sets (*p*_01_, *p*_02_), (*p*_01_, *Nmm*), and (*p*_02_, *Nmm*)), the results are depicted in Figures 5–7. These figures illustrate tumor structural details qualitatively on day 12th. Tables 7–9 show the tumor structures quantitatively for each set, respectively.

Regarding Table 7, the size of tumor in this regime (PGF), on day 12^th^, is around 60 to 61 percent of the considered tissue. The harmfulness measure of the tumor (GF) varies between 39 and 55 percent; in this condition, NF (the therapy effectiveness feature) is a number between 39 and 52 percent. This set of players has the worst results of fighting against cancer among the all considered sets, where it is highlighted in red color in Figure 5(a) and Table 7.

Figure 6 and Table 8 illustrate results of therapies in the BD group, where (*p*_01_, *Nmm*) are players. Although these players are a bit better than previous players of this group, but they should be categorized as weak players still.

Another set of players in the BD group is (*p*_02_, *Nmm*), where their play results against tumor growth are summarized in Figure 7 and Table 9. Results confirm that we may label these players as weak ones like the other players of the BD group.

### 3.3. Group CD Combined Therapies

In this approach of combination therapies, we assume that therapy actors disturb progrowth transmission and DNA replication process at the same time.

For investigating the behavior and properties of this kind of combination therapies, we do the same as the two previous groups. This strategy has three sets of two players: (age, *p*_01_), (age, *p*_02_), and (age, *Nmm*). It is noteworthy that the effects of players (age, *p*_01_) and (age, *p*_02_) have been investigated as players (*p*_01,_age) and (*p*_02_, age) in the BC group beforehand; therefore, we only consider the player set (age, *Nmm*) as the agents of the CD group.

**Figure 8:**
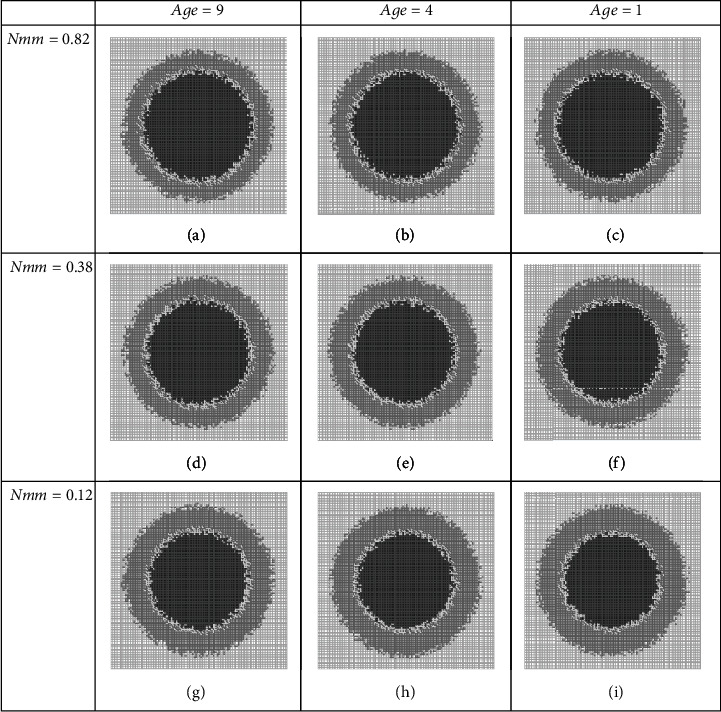
Pictures of tumor structures where targeting progrowth transmission and DNA replication process. All snapshots are on day 12, but at different dosages (a–i) of a combination therapy regime. Quantitative measures are listed in Table 10.

Qualitative and quantitative measures of therapies, when (age, *Nmm*) act against tumor growth, are summarized in Figure 8 and Table 10, respectively. It is seen that although these agents are better in comparison with the BD group teams, but are not as well as BC teams. The tumor size of 56% can be reached after 12 days of therapy, where the score of 40% of harmfulness will be realizable (the 52% of therapy effectiveness measure); NF can be accessible by this team.

## 4. Discussion

Precision medicine and combination therapies can improve the life expectancy of most patients and diminish damages to the tissues surrounding the tumor [36–38]. In this way, mathematical models and *in silico* experiments are of great help. In this research, we used ABSM and hypothesized some relations between the model parameters and anticancer drug (agents) groups; besides, some parameters were attributed implicitly to individual variability. We performed *in silico* experiments and investigated the effects of some combinations of these parameters as therapy players. Here, we analyze and discuss the postulated combination (and precision) therapy strategies and evaluate the results against recent findings in cancer biology and therapies.

As stated beforehand, one of the intents of the researchers in the field of oncology is to take into account individual variability for each person (e.g., in genes, environment, and lifestyle) and understand and examine potential role(s) of any combinations of therapies on cancer cells' growth and spread [10, 12, 14, 15, 39, 40]; in addition, with regard to cancer hallmarks named in Introduction, we see that six out of ten hallmarks are related to cell division explicitly or implicitly (hallmark numbers: 1, 2, 4, 6, 7, 9), and generally speaking (and setting aside environment and lifestyle), cancer is basically a genetic disease of cells; all of these mean that cell division probability (and cell cycle as well) is a genetic, i.e., individual representative.

A dose refers to a specified amount of medication (therapy) taken at one time, and dosage is the prescribed administration of a specific amount, number, and frequency of doses over a specific period of time, i.e., a dosage guides a drug regimen. Based on therapy type, doses are expressed in different metrics like mass units (e.g., milligrams), drops, and radiations.

**Figure 9:**
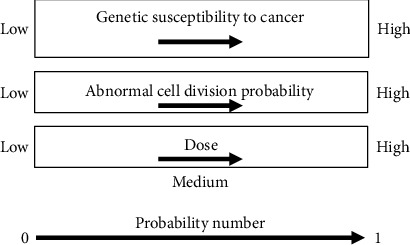
Schematic illustration of relations among (1) personalized genetic susceptibility to cancer, (2) probability of abnormal division of cells, (3) low, medium, and high dose quantity, and (4) probability number. In this research, we map all of the above named concepts to the [0, 1] interval. Note that this mapping can be complex and nonlinear.

**Figure 10:**
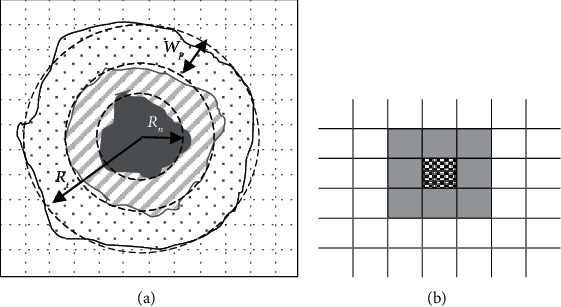
Physical structure of the ABSM as a square lattice. (a) The considered tumor is composed of three layers: necrotic cells (dark grey layer), quiescent cells (middle shaded layer), and proliferating cells (dotted layer). The average radius of the tumor, necrotic layer, and the average thickness of the outer proliferating cancerous cells layer are shown by *R_t_*, *R_n_*, and *W_p_*, respectively. (b) The Moore neighbors (grey cells) of the central (checker background) cell.

**Figure 14:**
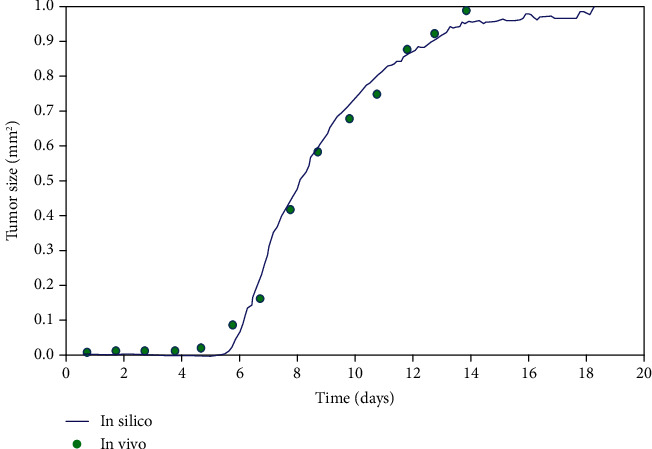
Validation of ABSM. The size of the simulated tumor (blue solid line) compared to the size of the tumor in BALB/c mice (green circles) that have been involved with CT26-TK cells [90].

However, to avoid a special kind of metric or therapy, we use more general metrics (*High*, *Medium*, and *Low* doses) as it is common in medicine. In this situation, a physician can have his or her own interpretation from High, Medium, and Low metrics according to underlying cases. In this way, we may attribute [0, 1] interval as a mathematical representation (or normalized quantity) of [Low, High] doses (and dosage as well). This issue is illustrated in Figure 9. Note that Figure 9 illustrates only the mapping between domains, and not the exact relations among variables of different domains; in fact, relationships may be complex and nonlinear. Nevertheless, “High, Medium, Low” approach can be considered as an alternative for avoiding engagements in complex relations among variables of different domains.

To evaluate the best combination therapy strategy, we not only used qualitative graphical structures of the tumors on day 12^th^ that are depicted in Figures 3–8 but also used quantitative measures (pure tumor growth fraction (PGF), growth fraction (GF), and necrotic fraction (NF)) [28]. PGF, that is the fraction of the whole number of tumorous cells to the whole number of all cells in tissue, *n*cell × *n*cell, can be treated as a quantitative measure of the tumor size; a less PGF on day 12^th^ means a smaller tumor.

GF, that is the fraction of the population value of tumor proliferative cells to the population value of all tumorous cells, can be treated as a measure of the aggression and invasion potential of tumor; therefore, if two tumors have the same size on day 12^th^, we should look at their malignancy and prognosis for their future growth; in this case, GF can be used as a measure; the more the value of GF, the more harmfulness and aggression can be expected.

NF, i.e., necrotic fraction, is the ratio of the number of necrotic cells to the whole number of tumorous cells; therefore, in spite of GF, NF can be treated as a measure of manageability and controllability of the tumor; a larger value of NF on day 12^th^ means a less dangerous tumor. NF can be treated as a measure that shows how a therapy can prevent tumor cells to be proliferative.

One of the main problems in the examining different combinations of drugs (agents) is the huge number of different alternatives [14], e.g., when trying to identify the best combination of 10 drugs at 3 doses (e.g., High, Medium, and Low doses), one will have to test 3^10^ combinations. In this research, by defining five new features, we introduce a general algorithm for prediction (or suggestion) of the number of possible more (less) effective combinations. The usefulness of this algorithm is that we can try the best expected combinations of therapies at first and consume the time and expenses in clinical trials.

It is well established in oncology that different obstructing mechanisms at the same time (compound therapy) might lead to the best results of tumor development prevention [37]. In this circumstance, we expect therapies with more diverse players, i.e., the sets with different players from different groups and parameters that have different interpretations from cancer biology point of view will create more successful pairs of fighters against cancer. In this way, because different physical dimensions usually represent different action mechanisms, we give a positive score to therapy teams which have players with different physical dimensions; to deal with this favorite score, we have introduced a novel metric (DPD) in our research.

To introduce our algorithm and attribute the above biological findings to combination sets, we reorganize Table 3 as depicted in Table 11 and add the following feature numbers to it: the number of different players of the considered target groups (DTG) in a set, the number of same players of the considered target groups (STG) in a set, the number of different mechanisms of parameter (DMP) actions, the number of same mechanism of the parameter (SMP) actions, and finally, let us give an additional positive score to the teams with diversity number of the physical dimension (DPD) of the players.

As an example for better description of Table 11, in the fourth row, the features of the combined group “CD” have been summarized, i.e., considered groups are C and D. Reconsidering Tables 2 and 3, we see that parameters in each three sets, (age, *p*_01_), (age, *p*_02_), (age, *Nmm*), are all from different target groups, so we attribute the number 2 to DTG feature of each set. We also see that in all three sets, (age, *p*_01_), (age, *p*_02_), (age, *Nmm*), there is no parameter in common between target groups C and D; therefore, we assign the number 0 to STG feature of each set. Regarding the definitions of each parameter in each three sets, (age, *p*_01_), (age, *p*_02_), (age, *Nmm*), it is clear that they affect the tumor growth by different mechanisms; therefore, we assign the number 2 to DMP feature of each set. As reasoned above, we should give the number 0 to SMP feature of each set. It is interesting to note that each parameter in each set of (age, *p*_01_), (age, *p*_02_), (age, *Nmm*) has different physical dimensions (time or probability); so as you see in the last column of Table 11, we may assign an additional positive score to each team, i.e., the number 2 to DPD feature of each set for the CD group.

Considering definition of terms, DTG, STG, DMP, SMP, and DPD, it is clear that in a considered therapy, the higher the value of a term that begins with letter D and the lower the value of a term that begins with letter S, the better feature that therapy has.

Inspecting Tables 5–10, we can find the best and the worst therapies as follows: the best therapy result belongs to the combined group BC, where *p*_01_ and age are treated as therapies agents; the tumor features of this therapy on day 12^th^ are highlighted in Table 5. The worst result can be assigned to the combined group BD, where *p*_01_  and *p*_02_ are therapies players; the tumor features of this therapy on day 12^th^ are highlighted in Table 7. The features of these two therapies are compared in Table 11. It is clear that *p*_01_ and age are players from different target groups (DTG = 2, STG = 0) and affect tumor growth with different mechanisms (SMP = 0) and having different physical dimensions (DPD = 2). It is where *p*_01_ and *p*_02_ are players that are common between target groups B and D (DTG = 0, STG = 2) and affect tumor growth with different mechanisms ( SMP = 0) and having same physical dimensions (DPD = 0).

In this research, the hypothesized algorithm has been discussed for the set of therapies with two players; nevertheless, it can be extended to three and more player sets in the future researches. In fact, the importance and meaning of presented quantitative merits (DTG, STG, DMP, SMP, and DPD) are more realizable, when one faces the sets with three or more players.

Although the presented research can help one to broaden the conclusions drawn from existing medical data, suggest new experiments, test hypotheses, predict behavior in experimentally unobservable situations, but has some limitations that need further investigations.

Where the value of a parameter as a therapy player may be controlled by dose value, some of parameters may also be related to individuals' genetic contents. In this context, although Table 5 represents the best therapy results, but it is seen that the best of the best results is where we set a high dose value to control *p*_01_ and an intermediate value for age; in this way, an interesting deduction from precision medicine point of view can be persons with intrinsic genome of low values *p*_01_ and intermediate values for age have less need for BC group therapies. Discussing the same with a look at Table 7, it is seen that the worst results are for high doses for controlling *p*_01_ and *p*_02_ when we deal with a given patient with personal characteristics like Table 15. The implementation of the roles of dose values in our presented algorithm should be more investigated.

Biological systems are complex; they are composed of several agents and sophisticated interactions (which look to be stochastic) among them. This is where stochastic system biology-based models can help us. In fact, in this circumstance, models such as our ABSM seems to be more compatible with the biology of cancer than ODE or PDE ones; in addition, although personalization and relationship between dosages and parameter values in ABSM look like to be difficult and a challenging issue, but proposed “High, Medium, Low” approach which is pharmacologically (biologically and therapeutically) plausible seems to be satisfactory; however, this matter should be more investigated in the future researches.

One of the current limitations of our model is that we have not considered angiogenesis in it, and therefore, it has no delegated parameters in the target group A. We are hoping to add angiogenesis to ABSM in our future work.

In this research, because the number of model parameters that are attributed to players of different target groups is limited, therefore, we could not investigate all possible combination sets of group players, e.g., BE, CE, and DE.

We think about making a training and teaching software tool, using our proposed model and method. The results of this work should be more explored from clinical applications' point of view, and our research colleagues in Cancer Institute of Iran are looking for more biological validations of our findings by implementing *in vitro* experiments.

An interesting direction for future research can be the investigation of side effects of each combined groups. A side effect is an important issue in cancer drug therapy [38]. It can decrease the life quality of a patient. It seems reasonable that combination therapies that can employ lower doses of each therapy element have the potential to milder side effects. It means that for more precious deductions from Tables 5–10, we may introduce a new merit that involves dose-dependent side effects of each combined groups. In addition, because side effect, i.e., damage to NA_0 agents due to drugs, is not involved in ABSM, we hope to implement the modified version of the model in the future.

Our colleagues in the Cancer Research Institute of Iran [41] are working on the setting up of statistical studies to test the proposed combination precision cancer therapy. However, although the quality of a scientific field depends on how well the mathematical descriptions developed on the theoretical side agree with results of experiments, possible lack of agreement between theoretical mathematical explanations and experimental measurements often leads to important advances as better theories are developed.

It should be noted that ABSM like other agent-based models may suffer from computational bottleneck for large numbers of cells [27]. This matter should be investigated, and possible use of “hybrid” approaches can be considered in the modified version of ABSM.

## 5. Conclusions

Treatment schedule and therapies strategies can improve the life expectancy of cancer patients.

Although precision medicine along with consolidation of cancer therapies, combination therapy enhances efficiency compared to the monotherapy approach, but which combination for which person is the most effective one is an open question. In this study, we presented a method and novel quantitative merits—DTG, STG, DMP, SMP, DPD—that an oncologist can use to estimate and predict effectiveness of therapies for each individual and make quantitative rankings among therapies. *In silico* experiments showed that combined therapies are more effective when players affect tumor via different mechanisms and have different physical dimensions.

## Figures and Tables

**Figure 11:**
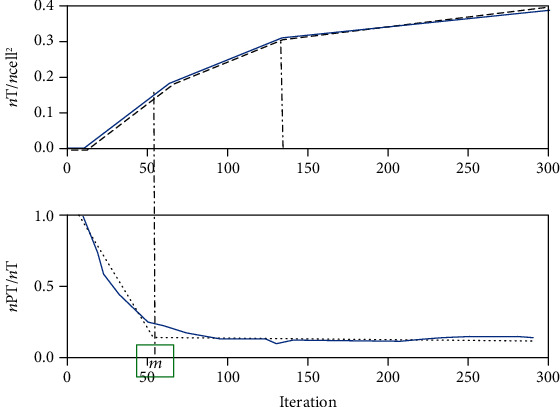
An example plot of PGF(*m*) (upper) and GF(*m*) (bottom) against iterations in a typical simulation. The formation and development of necrotic layer are manifested by tracking the changes in the slope of the curves. Evident change of the slope of the curve occurs at around the 60th iteration, and after that, random walk search by IAs is initiated.

**Figure 12:**
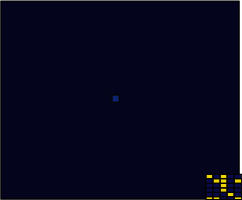
The initial condition for a typical simulation of the ABSM.

**Figure 13:**
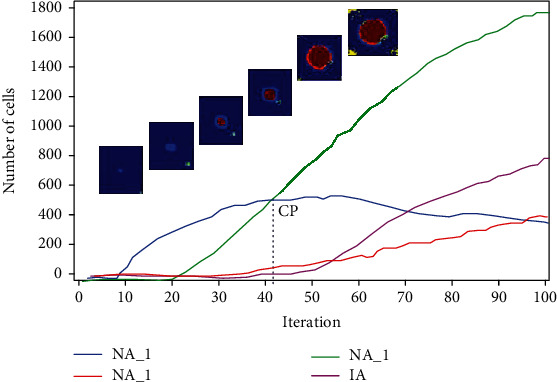
A typical simulation of ABSM. The number of NA_1, NA_2, NA_3, and IA vs. time. At critical point (CP), the number of NA_1 and NA_3 becomes equal, and NA_1 stops increment.

**Table 1:** Overview and brief descriptions of the ABSM elements [28]. The star components are those which hypothesized to be related to individual variability.

Element	Brief description
*n*T(*m*)	The whole number of tumor cells in the tissue at iteration *m*
*n*PT(*m*)	The whole number of NA_1 in the tissue at iteration *m*
*n*BI(*m*)	The number of IA newborns in the tissue at iteration *m*
*f*(*m*)	Sum of the numbers of failures in IA fighting against tumor cells till iteration *m*
*n*PT_*i*,*j*_	The number of NA_1s in the neighborhood of site (*i*, *j*)
*nI*_*i*,*j*_	The number of IAs in the neighborhood of site (*i*, *j*)
*R*_*t*_(*m*)	The average radius of the external edge of the tumor (Figure 10) at iteration *m*
*R*_*n*_(*m*)	The average radius of the necrotic zone (Figure 10) at iteration *m*
NF(*m*)	Necrotic fraction (Number of NA_−_3 at *m*/Number of whol tumor cells at *m*)
*ρ*_PT_(*i*, *j*, *m*)	The probability of producing a NA_1 in the site (*i*, *j*) at iteration *m*^∗^
Age	Maximum allowable time duration to stay in NA_1 mode without proliferation^∗^
*Nmm*	The production probability of a NA_1_2 from the mitosis of a NA_1_1^∗^
*v*(*m*)	A whole number of victories of IA in fighting against tumor cells at iteration *m*
*ρ*_*t*_	Protumor probability^∗^
*ρ*_*I*_	Antitumor probability^∗^
*W*_*p*_	NA_1 rim thickness
PGF(*m*)	Pure tumor growth fraction at iteration *m*
GF(*m*)	Growth fraction ((Number of NA_−_1 at *m*/Number of whol tumor cells at *m*))

**Table 2:** Summary of target groups in cancer therapies and hypothesized relations with ABSM's parameters.

Target group	Related parameters	Therapeutic type examples	Drug type examples
A		Monoclonal antibodies, chemotherapy/radiotherapy, small molecules	Bevacizumab [30], sunitinib [31]
B	*p*_01_,*p*_02_	Monoclonal antibodies, endocrine therapy	Trastuzumab [32], tamoxifen [33]
C	Age	Small molecules	Erlotinib [34]
D	*p*_01_,*p*_02_, *Nmm*	Chemotherapy/radiotherapy	Different types
E	*p*_01_, *p*_02_	Chemotherapy/radiotherapy	Different types

**Table 3:** Hypothesized combination therapies designed by ABSM parameters.

Combined groups	Combined parameters
BC	(*p*_01_,age), (*p*_02_,age)
BD	(*p*_01_, *p*_02_), (*p*_01_, *Nmm*), (*p*_02_, *Nmm*)
BE	Repeated; same as a set in BD
CD	(age, *p*_01_), (age, *p*_02_), (age, *Nmm*)
CE	Repeated; like BC
DE	Repeated; like BD

**Table 4:** Parameters values used in *in silico* experiments.

Parameter	High value	Medium value	Low value
*p*_01_	0.81	0.52	0.23
*p*_02_	0.75	0.42	0.26
Age	9	4	1
*Nmm*	0.82	0.38	0.12

**Table 5:** Quantitative comparison of the results of BC group therapies, where *p*_01_ and age are therapy agents.

Individual characteristics	Dose value	H 0.81	M 0.52	L 0.23
Age = 1, *p*_01_ = 0.7	H 9	PGF = 0.39,GF = 0.44, NF = 0.48	PGF = 0.39, GF = 0.53, NF = 0.39	PGF = 0.40, GF = 0.55, NF = 0.37
	M 4	**P** **G** **F** = 0.38, **GF** = 0.40, **NF** = 0.52,	PGF = 0.39, GF = 0.51, NF = 0.41	PGF = 0.39, GF = 0.41, NF = 0.50
	L 1	PGF = 0.40, GF = 0.47, NF = 0.44	PGF = 0.39, GF = 0.50, NF = 0.42	PGF = 0.39, GF = 0.46, NF = 0.46

First column: two considered personalize characteristics. Second column: dose value for controlling first characteristic. First row: normalized dose values for controlling 2nd characteristic.

**Table 6:** Quantitative comparison of the results of BC group therapies, where *p*_02_ and age are therapy agents.

Individual characteristics	Dose value	H 0.81	M 0.52	L 0.23
Age = 1, *p*_02_ = 0.5	H 9	PGF = 0.51, GF = 0.43, NF = 0.48	PGF = 0.51, GF = 0.53, NF = 0.40	PGF = 0.51, GF = 0.54, NF = 0.38
	M 4	PGF = 0.51, GF = 0.40, NF = 0.51	PGF = 0.51, GF = 0.51, NF = 0.41	PGF = 0.51, GF = 0.42, NF = 0.49
	L 1	PGF = 0.51, GF = 0.47, NF = 0.44	PGF = 0.51, GF = 0.49, NF = 0.43	PGF = 0.51, GF = 0.45, NF = 0.47

First column: two considered personalize characteristics. Second column: dose value for controlling first characteristic. First row: normalized dose value for controlling 2nd characteristic.

**Table 7:** Quantitative comparison of the results of BD group therapies, where *p*_01_ and *p*_02_ are therapy agents.

Individual characteristics	Dose value	H 0.81	M 0.52	L 0.23
*p*_01_ = 0.7, *p*_02_ = 0.5	H 0.75	**P** **G** **F** = 0.61, **GF** = 0.55, **NF** = 0.39	PGF = 0.60, GF = 0.52, NF = 0.40	PGF = 0.60, GF = 0.47, NF = 0.45
	M 0.42	PGF = 0.60, GF = 0.50, NF = 0.42	PGF = 0.60, GF = 0.42, NF = 0.50	PGF = 0.60, GF = 0.45, NF = 0.47
	L 0.26	PGF = 0.60, GF = 0.49, NF = 0.43	PGF = 0.60, GF = 0.39, NF = 0.52	PGF = 0.60, GF = 0.43, NF = 0.49

First column: considered personalize characteristics. Second column: normalized dose value for controlling first characteristic. First row: normalized dose value for controlling 2nd characteristic.

**Table 8:** Quantitative comparison of the results of BD group therapies, where *p*_01_ and *Nmm* are therapy agents.

Individual characteristics	Dose value	H 0.81	M 0.52	L 0.23
*p*_01_ = 0.7, *Nmm* = 0.2	H 0.82	PGF = 0.60, GF = 0.43, NF = 0.49	PGF = 0.60, GF = 0.49, NF = 0.43	PGF = 0.60, GF = 0.54, NF = 0.39
	M 0.38	PGF = 0.60, GF = 0.42, NF = 0.50	PGF = 0.60, GF = 0.47, NF = 0.45	PGF = 0.60, GF = 0.52, NF = 0.40
	L 0.12	PGF = 0.60, GF = 0.39, NF = 0.52	PGF = 0.60, GF = 0.45, NF = 0.47	PGF = 0.60, GF = 0.50, NF = 0.42

First column: considered personalize characteristics. Second column: normalized dose value for controlling first characteristic. First row: normalized dose value for controlling 2nd characteristic.

**Table 9:** Quantitative comparison of the results of BD group therapies, where *p*_02_ and *Nmm* are therapy agents.

Individual characteristics	Dose value	H 0.75	M 0.42	L 0.26
*p*_02_ = 0.5, *Nmm* = 0.2	H 0.82	PGF = 0.60, GF = 0.49, NF = 0.43	PGF = 0.60, GF = 0.43, NF = 0.49	PGF = 0.60, GF = 0.54, NF = 0.39
	M 0.38	PGF = 0.60, GF = 0.45, NF = 0.47	PGF = 0.60, GF = 0.39, NF = 0.52	PGF = 0.60, GF = 0.50, NF = 0.42
	L 0.12	PGF = 0.60, GF = 0.47, NF = 0.45	PGF = 0.60, GF = 0.42, NF = 0.50	PGF = 0.60, GF = 0.52, NF = 0.40

First column; considered personalize characteristics. Second column; normalized dose value for controlling first characteristic. First row; normalized dose value for controlling 2nd characteristic.

**Table 10:** Quantitative comparison of the results of CD group therapies, where age and *Nmm* are therapy agents.

Individual characteristics	Dose value	H 9	M 4	L 1
*Nmm* = 0.2, Age = 1	H 0.82	PGF = 0.56, GF = 0.40, NF = 0.52	PGF = 0.56, GF = 0.41, NF = 0.50	PGF = 0.56, GF = 0.43, NF = 0.48
	M 0.38	PGF = 0.56, GF = 0.45, NF = 0.47	PGF = 0.56, GF = 0.47, NF = 0.44	PGF = 0.56, GF = 0.49, NF = 0.44
	L 0.12	PGF = 0.56, GF = 0.50, NF = 0.42	PGF = 0.56, GF = 0.53, NF = 0.40	PGF = 0.56, GF = 0.54, NF = 0.38

First column: considered personalize characteristics. Second column: normalized dose value for controlling first characteristic. First row: normalized dose value for controlling 2nd characteristic.

**Table 11:** Combination therapy sets and their associated features.

Combined groups	Combined parameters	DTG	STG	DMP	SMP	DPD
BC	(*p*_01_, age), (*p*_02_, age)	2, 2	0, 0	2, 2	0, 0	2, 2
BD	(*p*_01_, *p*_02_), (*p*_01_, *Nmm*), (*p*_02_, *Nmm*)	0, 1, 1	2, 1, 1	1, 1, 1	1, 1, 1	0, 0, 0
BE	Repeated; like a set in BD	—	—	—	—	—
CD	(age, *p*_01_), (age, *p*_02_), (age, *Nmm*)	2, 2, 2	0, 0, 0	2, 2, 2	0, 0, 0	2, 2, 2
CE	Repeated; like BC	—	—	—	—	—
DE	Repeated; like BD	—	—	—	—	—

**Table 12:** Introduction of agents of ABSM and their states.

Agent	Description	Possible states	Symbol
NA	Nonimmune cells	0—normal cell or empty space (N)	NA_0
		1—proliferating tumor cell (PT)	NA_1
		2—nonproliferating tumor cell or quiescent cell (NT)	NA_2
		3—necrotic cell (Ne)	NA_3
IA	Immune cells	0—natural killer cell	IA_0
		1—cytotoxic T lymphocyte (CTL)	IA_1

**Table 13:** Hypothesized relations among model parameters and cancer hallmarks and TME players. Star parameters are assumed to be individuals' genetic, molecular, or cellular dependency.

Parameter	Representative hallmark	Hypothesized TME player
^∗^ *p*_01_	1, 2, 4, 6, 7, 8, 9	1, 2
^∗^ *p*_02_	1, 2, 4, 6, 7, 8, 9	1
^∗^ *K*_*dT*_	8	2, 6, 5
^∗^ *K*_*dI*_	1, 2, 3	2, 6, 4, 5
^∗^ *Nmm*	1, 2, 4, 6, 9	6
*R*_max_	7	6
*a*	1, 2	6
*b*	1, 2	6
^∗^Age	1, 2, 4, 6, 9	6

**Table 14:** The typical value of ABSM parameters used in simulations.

Parameter	Brief explanation	Value
*p*_01_	Base probability of division of NA_1_1 cell	0.7
*p*_02_	Base probability of division of NA_1_2 cell	0.6
*a*	Base necrotic thickness, controlled by nutritional needs	0.42 [30, 89]
*b*	Base proliferative thickness, controlled by nutritional needs	0.11 [30, 89]
*R*_max_	Maximum tumor extent, controlled by pressure response	37.5 mm [30, 89]
*K*_*dT*_	Tumor death constant	0.5
*K*_*dI*_	Immune death constant	0.2

**Table 15:** The parameter values of the ABSM for reproducing *in vivo* data reported in [90].

Parameters	Values
*p*_01_	0.7
*p*_02_	0.5
*a*	0.42
*b*	0.11
*R*_max_	15 mm
*K*_*dT*_	0.5
*K*_*dI*_	0.2
*Nmm*	0.2
Age	1

## Data Availability

No data were used to support this study.

## Appendix

Our ABSM is founded on four bases: (1) biological assumptions, (2) physical structure, (3) agents and their states, and (4) states transition rules.

In ABSM, host tissue is assumed as a two-dimensional lattice composed of *n*cell × *n*cell squares as illustrated in Figure 10. Here, each square of the lattice is called a cell.

As is shown in Figure 10, we assume that a tumor consists of three layers: (1) the outer layer of proliferative tumor cells (dotted region), (2) the middle layer of nonproliferating or quiescent tumor cells [34] (dashed grey region), and (3) the inner layer of necrotic cells (dark grey core). In the ABSM, with the physical neighborhood, we mean Moore neighborhood as is depicted in Figure 10(b).

### A. Agents and Their States

In the ABSM, two types of agents (immune and nonimmune cells) are presumed. Definitions of agents and their states are summarized in Table 12.

Each agent represents a biological cell placed at (*i*, *j*) coordinate, where 0 < *i*, *j* < *n* − cell.

The IA is a moving agent and may accidentally collide with NAs. In this paper with the total number of tumor cells, we mean the sum of NA_1, NA_2, and NA_3. In the ABSM, it is supposed that each IA has the ability to call other immune cells.

In ABSM, NA_0 cells play the role of empty places for NA_1 or IA cells. For example, the IA cells that are looking for cancer cells or recalled to a specific location may substitute the NA_0 cells, i.e., possess NA_0 sites.

To our knowledge from cancer biology, if NA_1 cells are surrounded by more healthy cells, they will access more nutrients and oxygen; it means that the higher is the number of healthy cells, NA_0 (empty space) around a proliferative tumor cell (NA_1), the greater is the likelihood of division of the NA_1 cell. Two types of PT cells with different division probabilities are considered. In the first type denoted by NA_1_1, the cell division is assumed independent from the environmental conditions, i.e., the division probability (*ρ*_PT_ is constant), while in the second type is symbolized by NA_1_2, i.e., the cell division probability (*ρ*_PT_ is a function of its healthy neighbors (NA_0s)).

In the ABSM, it is assumed that each NA_1_1 may be divided into one NA_1_1 and one NA_1_2 daughter cells with the probability *Nmm* and into two NA_1_1 daughter cells with the probability (1 − *Nmm*). However, it is assumed that each NA_1_2 cell can only be divided into two NA_1_2 daughter cells.

At every simulation time step, the chance of each NA_1 (either NA_1_1 or NA_1_2) for proliferation (division) is checked, i.e., the existence of at least one empty place in the neighborhood of that parent cell is checked. If there is at least one empty space (NA_0) in its neighborhood, the NA_1 cell will divide with the probability *ρ*_PT_ into two NA_1 daughter cells, so that one of the daughter cells will stay in the position of her parent and the other daughter will place in that empty neighbor. If there is more than one empty place in the neighborhood, the second NA_1 daughter cell will pick one of the empty spaces randomly (with the same probability). If the NA_1 cell cannot find any empty place in its neighborhood or if it cannot proliferate (with the probability 1 − *ρ*_PT_), it would stay at NA_1 state. The maximum of time length at which NA_1 can stay without any division is limited to age. If NA_1 has no chance for proliferation for a time length (age), then its state will be changed to nonproliferating (quiescence) tumor (NA_2).

Researches indicate that there are many levels of quiescence. Various signals such as serum starvation or loss of nutrients, loss of adhesion, and cell contact inhibition at high cell density can induce quiescence to a tumor cell [42–44]. In the ABSM, the above fact has been considered by introducing the lifetime parameter age.

If a NA_2 cell is placed at a radial distance less than the radius of the necrotic core (*R*_*n*_ in Figure 10), then it will turn to NA_3 (necrotic cell) at the next time step.

If a NA_2 cell is at a radial distance less than *W*_*p*_ from the external edge of the tumor (see Figure 10), it may turn to a NA_1 cell due to accessing nutrient.

The NA_3 cells accumulate in the inner zone of the tumor and form a necrotic layer. Their state will not ever change to any other states since they are already dead.

Researchers have found that tumor cells release signals [43] in the host tissue which can be detected by immune cells via specific receptors. The immune cells will receive these signals and search for the tumor cells. For more compatibility of the ABSM with cancer biology, we introduced pure tumor growth fraction (PGF(*m*)) and growth fraction (GF(*m*)) concepts. PGF(*m*) refers to the ratio of the total number of tumor cells at time *m* to the total number of cells in the studied tissue (Equation (A.1)). GF(*m*) denotes to the fraction of the number of NA_1 cells (NA_1_1 + NA_1_2) to the total number of tumor cells at time *m* (Equation (A.2)).

(A.1) $$\begin{array}{c} \text{PGF}\left(m\right)=\frac{n\text{T}\left(m\right)}{{n\text{cell}}^{2}\;}, \end{array}$$

(A.2) $$\begin{array}{c} \text{GF}\left(m\right)=\frac{n\text{PT}\left(m\right)}{n\text{T}\left(m\right)}. \end{array}$$

At the start of the tumor growth in a tissue, when PGF(*m*) is negligible, IAs enter the tissue from one corner of the lattice Figure 10, where they have long distance from the center of the tumor. In this circumstance, each IA is assumed to move directly towards the center of the tumor and will take the place of an empty space in its Moore neighborhood. This empty space neighbor is one that has the shortest Euclidean distance from the center of the tumor.

Figure 11 shows a typical simulation result of the ABSM. It can be seen that at time *m* ≥ 60, the growth fraction factor (GF(*m*)) remains almost constant, which means that the necrotic layer is formed and is noticeable. After this time, the IAs can walk randomly in any direction to meet tumor cells.

In the ABSM, we implement short-range or contact-dependent biological communication [44]. It means that any interaction between IA and NA agents takes place only when they are in physical contact, i.e., when they are neighbors. When an IA encounters an NA, one of the following events may take place:

(1) *Antitumor Action*. In this case, the NA will fail. If IA_1 encounters the NA_1, the NA_1 cell may die with the probability *ρ*_*I*_ (Equation (A.3)) and will change to NA_0 state at the next iteration
  (A.3) $$\begin{array}{c} p_{I}=K_{dT}\times \frac{{nI}_{i,j}}{{n\text{PT}}_{i,j}}. \end{array}$$

In Equation (A.3), *K*_*dT*_ is tumor death constant; *nI*_*i*,*j*_ and *n*PT_*i*,*j*_ are the numbers of IA_1 cells and NA_1 cells, respectively, in the neighborhood of the studied cell (cell located at position (*i*, *j*)).

In other words, the IA_1 will substitute the NA_1 which is located in its neighborhood with the probability *K*_*dT*_, and the NA_1 will be dismissed from the microenvironment. If there are more than one NA_1 in the neighborhood of the IA_1, the IA_1 will kill all NA_1 cells and randomly replace one of them. The other NA_1 cells will change into NA_0.

If the collision agent is IA_0, the appropriate rules will apply only to one of the randomly chosen (with a uniform density function) NA_1 cells in its neighborhood.

(2) *Null Action*. In this case, nothing happens, i.e., both agents; NA_1 and IA will survive and remain in their states. Meanwhile, the IA will continue its search to find another NA_1
(3) *Protumor Action*. In this case, the IA will fail in killing NA_1 and die with probability *ρ*_*t*_ (Equation (A.4)). The IA will become an empty space, i.e., the IA will be removed from the tissue and will be replaced by a NA_0
  (A.4) $$\begin{array}{c} \rho_{t}=K_{dI}\times \frac{{n\text{PT}}_{i,j}}{{nI}_{i,j}}. \end{array}$$

In Equation (A.4), *K*_*dI*_ is the immune death constant; *nI*_*i*,*j*_ and *n*PT_*i*,*j*_ are as mentioned earlier in Equation (A.3).

In the ABSM, in consistency with cancer biology, the ability to recruit immune cells and birth of new ones is considered. The number of newly born cells in each time step (*n*BI(*m*)) in Equation (A.5) is assumed as a function of the number of victories (*v*_*m*−1_) and the number of failures (*f*_*m*−1_) in the destruction of the tumor cells at time *m* − 1:

(A.5) $$\begin{array}{c} n\text{BI}\left(m\right)=\left(v\left(m-1\right)-f\left(m-1\right)\right)\times \frac{n\text{PT}\left(m\right)}{n\text{T}\left(m\right)}, \end{array}$$

where *n*T(*m*) and *n*PT(*m*) are the sum of the number of tumor cells and the total number of NA_1 cells, respectively, in the studied tissue at time *m*.

In fact, the more immune cells are successful to fight against tumor cells (*v* − *f* > 0), the more will be recalled. However, if *v* − *f* < 0, there will be no newborn immune cell in the studied tissue. The newborn immune cells will enter from the four corners of the lattice into the tissue randomly. The transition rules of IA will be applied to each of the newborn IAs.

The basic ABSM elements and their brief descriptions are summarized in Table 1. As it is deduced from Table 1 and above discussions, the ABSM is a system biology oriented one, i.e., it is constructed from several agents and interactions rules between them and as we will see these elements and interactions can give rise to the function and behavior of cancer growth system. With a conceptual and intuitive look at the elements of Table 1, some of them can be assumed individual-dependent; those are indicated by a star mark.

For better realization of our model, we hypothesize possible connections of each parameter to cancer hallmarks and TME follows. The hypothesized relations that are based on biological findings and arguments are listed in Table 13. In this table, the number of hallmarks or TMEs is same as the number that was given to them in Introduction. For example, in the first and second columns of the first row of Table 13, we have listed the possible effect of the valve of parameter *p*_01_ in cancer hallmarks and possible TME players which are delegated by *p*_01_; here, the number 1 means that we hypothesize that parameter *p*_01_ will influence the hallmark number 1 (unlimited multiplication) and TME player number 1 (fibroblasts and myofibroblasts) as well. Here, our approach is based on our interpretations from oncology literature, following discussions, and *in silico* experiments.

As we mentioned earlier, fibroblasts and myofibroblasts are the number one component of TME. Researches have confirmed that only the activated fibroblasts are required to initiate and endorse tumor growth [44–47]. When the fibroblasts remain activated after the initial insult has regressed, these activated fibroblasts may work with other molecular pathways to boost neoplasm initiation, where it has a significant impact on cancer progression through remodeling ECM, inducing angiogenesis, employing inflammatory cells, and directly stimulating cancer cell proliferation via the secretion of growth factors, immune suppressive cytokines, and mesenchymal-epithelial cell interactions [48, 49].

Experimental evidences prove that neuroendocrine cells, the number two component of TME, exhibit a combination of neuronal and endocrine features [50]. Neuroendocrine cells are the accomplices of tumor formation [51]. It is proven that the neuroendocrine system strongly influences the function of the immune system. Neuroendocrine cells could stimulate the proliferation of prostate carcinoma cells and increase their aggressiveness [52], while in the development of neuroendocrine cell tumors, they may play a principal role [53].

Some features of adipose tissue, the number three component of TME, are associated with cancer. Adipose cells secrete more than 50 different cytokines, chemokines, and hormone-like factors [54, 55]. These factors, whose production may upregulate in obesity, may be assistants in tumor initiation. Obese adipose tissue hypoxia establishes a highly proinflammatory microenvironment, which is more likely to breed tumors [56–58]. Adipose tissue reprogramming and the associated systemic secretion may have an effect on cancer growth and progression [59]. Excess adiposity leads to high circulating blood estrogen [60] and chronic, low-grade inflammation, which is involved in cancer development, a major energy source, relating with inflammation, recruiting immune cells, and supporting vasculogenesis [54, 61, 64].

The mammalian immune system monitors tissue homeostasis to protect against invading infectious pathogens and to eliminate damaged cells [62]. Findings recommend that immune surveillance has significant roles in recognizing and eradicating a large part of emerging tumor cells [2]. However, unlike normal functions, immune inflammatory cells, the number four component of TME, would persist in sites of chronic inflammation, linked to diverse tissue pathologies, including fibrosis, aberrant angiogenesis, and neoplasia [63]. Recent discoveries in immune system research illuminate that it is difficult to ignore the crucial issue that immune inflammatory cells may be the early support structure of cancer, having a double effect on tumor formation [64–70].

Blood and lymphatic vascular networks, the number five component of TME, help tumor cells escape immune surveillance in the process of tumor progression. Escape measures are mainly divided into two categories. Directly, the lymphatic microenvironment will weaken or eliminate the normal function of immune cells. For instance, the myeloid-derived suppressor cells could restrict the normal operation of T cells [71–73]. When the metastatic tumor enters a new environment, CD4^+^and CD8^+^ T cells may help them to evade the host immune system [74, 75]. Indirectly, the remodeling of unusual endothelial venules can influence immune cells to move into the lymph nodes [76].

ECM, the number six component of TME, is a dynamic and complicated environment. Evidences show that ECM can help neoplasms to get away from immune surveillance [77]. ECM contains all the cytokines, growth factors, and hormones secreted by stromal and tumor cells. A model indicated that ECM heterogeneity is crucial for controlling collective cell-invasive behaviors and determining metastasis efficiency [78–87]. ECM may affect tumors through extracellular secretion. In addition, ECM may alter the phenotype type of stromal cells or tumor cells [88]. The ECM of tumor will provide a hypoxic or acidic environment in which the tumor cells have greater survival advantages than normal cells. ECM will select survival cells to aid in tumor growth and invasion at the fastest rate.

### B. ABSM Evaluation

Using MATLAB codes, the ABSM was implemented.

In following simulations, at first, it is supposed that the entire tissue (lattice in Figure 10) is covered with NA_0s. The state of one cell in the center of the tissue is changed into NA_1 at time *m* = 0. The 0.1% of total number of cells (i.e., 0.001 × *n*cell × *n*cell), where *n*cell = 100, in the studied tissue, are entered as IAs from a corner of the lattice randomly. Figure 12 shows the initial condition for a typical simulation, where Table 14 depicts typical value of parameters.

The ABSM was evaluated qualitatively and quantitatively considering the following criteria: the average radius of the external edge of tumor at iteration *m*(*R*_*t*_(*m*)), the average radius of the necrotic zone at iteration *m*(*R*_*n*_(*m*)), growth fraction at iteration *m* (GF(*m*)), necrotic fraction at iteration *m* (NF(*m*)), and the number of NA_1, NA_2, NA_3, and IA cells at iteration *m*.

Besides, the graphical illustration of the growth is used as qualitative evaluation in some cases.

Figure 13 shows the number of NA_1, NA_2, NA_3, and IA vs. iterations. It is seen that the number of IA enhances due to recruitment which is qualitatively consistent with cancer biology. In this simulation, although IA cells enter the tissue to fight against tumor cells, but the permanent presence of 400 cells of NA_1 in tissue is observed.

Figure 14 compares the size of the simulated tumor growth using the ABSM to an *in vivo* experimental result [90], where the model parameters are as Table 15. The reported *in vivo* data is for BALB/c mice that have been involved with CT26-TK cells. The sizes were normalized in order to check the results more accurately. The simulation starts at time *m* = 6 and iteration time step is 2-hours. It is seen that the simulation results using the ABSM are consistent with the reported *in vivo* result, that is, the ABSM can quantitatively simulate a real tumor growth.

Quantitative measures are listed in Table 5.

Quantitative measures are listed in Table 8.
